# A Synopsis of the Associations of Oxidative Stress, ROS, and Antioxidants with Diabetes Mellitus

**DOI:** 10.3390/antiox11102003

**Published:** 2022-10-10

**Authors:** Homer S. Black

**Affiliations:** Department of Dermatology, Baylor College of Medicine, Houston, TX 77030, USA; hblack@bcm.edu

**Keywords:** diabetes mellitus, oxidative stress, ROS, antioxidants, metabolic syndrome

## Abstract

The Greek physician, Aretaios, coined the term “diabetes” in the 1st Century A.D. “Mellitus” arose from the observation that the urine exhibits a sweetness due to its elevated glucose levels. Diabetes mellitus (DM) accounted for 6.7 million deaths globally in 2021 with expenditures of USD 966 billion. Mortality is predicted to rise nearly 10-fold by 2030. Oxidative stress, an imbalance between the generation and removal of reactive oxygen species (ROS), is implicated in the pathophysiology of diabetes. Whereas ROS are generated in euglycemic, natural insulin-regulated glucose metabolism, levels are regulated by factors that regulate cellular respiration, e.g., the availability of NAD-linked substrates, succinate, and oxygen; and antioxidant enzymes that maintain the cellular redox balance. Only about 1–2% of total oxygen consumption results in the formation of superoxide anion and hydrogen peroxide under normal reduced conditions. However, under hyperglycemic conditions, about 10% of the respiratory oxygen consumed may be lost as free radicals. Under hyperglycemic conditions, the two-reaction polyol pathway is activated. Nearly 30% of blood glucose can flux through this pathway—a major path contributing to NADH/NAD^+^ redox imbalance. Under these conditions, protein glycation and lipid peroxidation increase, and inflammatory cytokines are formed, leading to the further formation of ROS. As mitochondria are the major site of intracellular ROS, these organelles are subject to the deleterious effects of ROS themselves and eventually become dysfunctional—a milestone in Metabolic Syndrome (MetS) of which insulin resistance and diabetes predispose to cardiovascular disease.

## 1. Introduction

Although the Egyptians were aware of the disease nearly 3500 years earlier, it was the Greek physician, Aretaios (1st Century A.D.) who coined the term “diabetes” and was the first to describe the disease as an entity [1,2,3]. The term “mellitus” arose from the observation that the urine exhibits a sweetness resulting from its elevated glucose concentration [1,2,4]. This disease, diabetes mellitus (DM), accounted for 6.7 million deaths globally in 2021 as reported by the IDF Diabetes Atlas, 10th edition [5]. In the U.S., 37.3 million people suffer from DM, incurring an annual cost of $379 billion USD [6]. This disease results when the body does not synthesize adequate insulin, the hormone that facilitates the transport of blood sugar into cells where it is metabolized and converted to energy or does not respond to insulin sufficiently [6]. The Centers for Disease Control (CDC) in the U.S. lists three main types of diabetes [6]. Type I diabetes results from autoimmune destruction of beta-cells (pancreatic insulin-producing cells). The American Diabetes Association [7] also lists non-autoimmune idiopathic diabetes that has no known etiology and is strongly inherited. This is a Type I diabetes that requires insulin therapy for survival. Type II diabetes (T2DM) is due to insulin secretary defect, “insulin resistance” or insensitivity. T2DM is the major form of diabetes affecting about 90–95% of people with diabetes in the U.S. [2]. The CDC lists gestational diabetes as a third type. This type develops in pregnant women who have not been previously diagnosed as diabetic, and although the disorder usually subsides after the baby’s birth, both mother and child are predisposed to T2DM diabetes later in life [7].

It was demonstrated, almost 90 years ago, that many diabetic patients were “insulin insensitive”, and it was suggested that patients should be classified as insulin sensitive (later classified as Type 1 DM) and insulin insensitive or non-insulin-dependent diabetes (classified as Type 2 DM) [8]. Extensive research has shown that common to insulin resistance, a number of physiologic variables including hyperglycemia, hyperinsulinemia, increased plasma VLDL, higher triglycerides, decreased plasma HDL-cholesterol and high blood pressure increased the risks of T2DM and cardiovascular disease (CVD) [9]. This variable cluster of physiologic abnormalities was given the term *Syndrome X* [10,11]. Indeed, as it became clear that CVD risks, e.g., insulin resistance/hyperglycemia, obesity, dyslipidemia and hypertension, were not independent of one another and shared underlying and interrelated mechanisms and pathways, they were considered as a “syndrome” and given the term *Metabolic Syndrome* (MetS) [12]. The World Health Organization (WHO) defined the term in 1998 after consultations with experts in DM research [13] and required the presence of DM [14]. One problem that arose with the WHO definition of MetS was that all risk factors were weighed equally, although the underlying pathophysiology was thought to be related to insulin resistance. Consequently, various groups developed their own version of the definition, and some even questioned whether the clustering of risk factors should be considered a “syndrome” [15]. Comparisons of the definitions for MetS from the WHO, the National Cholesterol Education Program Adult Treatment Panel III (NCEP ATP III) and the International Diabetes Federation (IDF) show considerable variability in the selection and parameters of risk factors, although all allow the inclusion of diabetic patients [15]. Further complication is introduced when some risk factors must be adjusted for specific ethnic populations and country-specific cut-points [16]. Overall, MetS is defined as a cluster of metabolic high-risk factors, including T2DM, high blood pressure, dyslipidemia, elevated LDL cholesterol, low HDL, and elevated triglycerides—which are all conditions found in T2DM resulting from *insulin resistance*. “*When diabetes becomes clinically apparent, CVD risk rises sharply*” [17].

## 2. Oxidative Stress, ROS, and Antioxidants

Oxidative stress, via the production of Reactive Oxygen Species (ROS) and Reactive Nitrogen Species (RNS), is characterized by an imbalance between the generation and removal of these species. This redox imbalance has been proposed as an etiologic factor involved in insulin resistance, beta-cell dysfunction, and impaired glucose intolerance that ultimately results in T2DM [18]. Oxidative stress can arise from excess food intake and a sedentary lifestyle. Indeed, it has been shown that an intake of excessive calories can lead to a 5–10-fold increase in ROS that escape from normal respiratory chain regulation [19]. Moreover, evidence has been obtained from T2DM patients that demonstrate increased oxidative stress in response to post-prandial hyperglycemia [20].

## 3. ROS Formation

**Figure 1:**
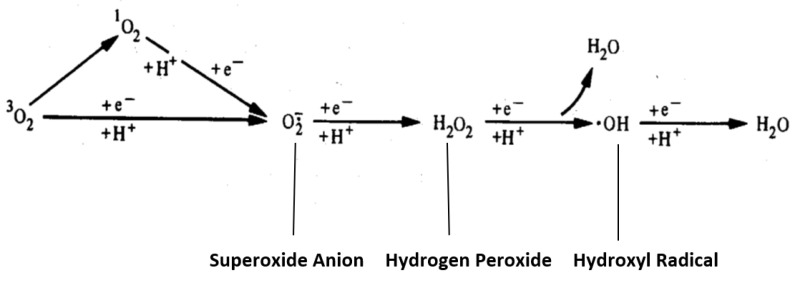
Reductive Pathway of Molecular Oxygen. An important alternate step is shown where an excited state species, singlet oxygen, is produced either via the interaction of molecular oxygen with the excited triplet state of another molecule or an energy exchange reaction upon the absorption of energy from another source, e.g., ultraviolet light [22].

Super-oxide anion is the primary radical formed by the univalent reduction in molecular oxygen. Subsequent reductions from super oxide anion to hydrogen peroxide and hydroxyl ion are spin-forbidden, and the non-enzymatic reactions proceed very slowly unless catalyzed by a heavy ion such as the metal catalyzed Haber/Weiss cycle [21]. These reactions are depicted in Figure 1.

**Figure 2:**
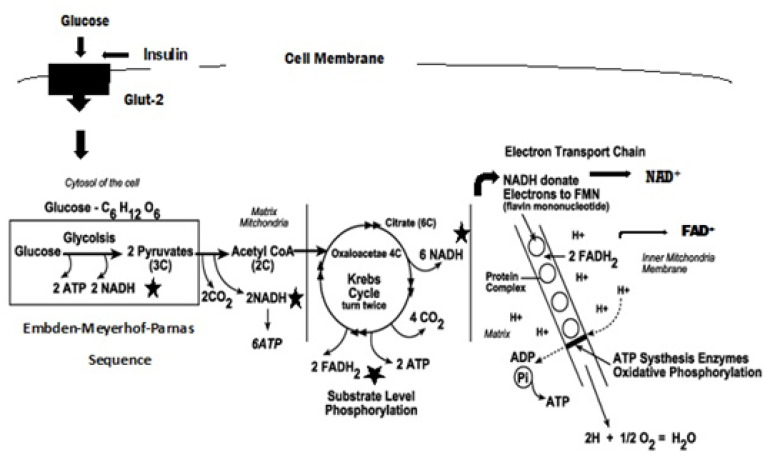
Euglycemic, normal insulin-regulated glucose metabolism. ★ Sites in the respiratory sequence at which low levels of superoxide may be formed through the transfer of electrons via the electron transport chain (ETC). Once glucose moves into the cell, it is retained though phosphorylation by hexokinase and ATP. The product, glucose-6-phosphate, is phosphorylated again to form fructose-1,6-diphosphare prior to splitting into 3-carbon fragments. These 3-carbon fragments are converted to pyruvic acid. All these reactions occur in the cell cytosol. Pyruvic acid enters the mitochondrial matrix and is decarboxylated and oxidized to the 2-carbon, acetyl CoA. This oxidization results in the formation of 2 NADH and the potential formation of low levels of superoxide as the electrons are passed through the ETC. Acetyl CoA enters the Krebs cycle by condensation onto oxaloacetate to form citrate that is decarboxylated and oxidized in the Krebs cycle to form 6 NADH and 2 FADH. These are potential ROS sources as the electrons are passed through the ETC. Flavin mononucleotide (FMN) is a highly oxidative cofactor associated with NADH and FADH dehydrogenases that can accept two electrons to become reduced. The figure depicts GLUT-2, an intestinal glucose transporter that allows effective glucose transport at high glucose concentrations. There are now a wide range of glucose transporters that are tissue specific [23].

**Figure 3:**
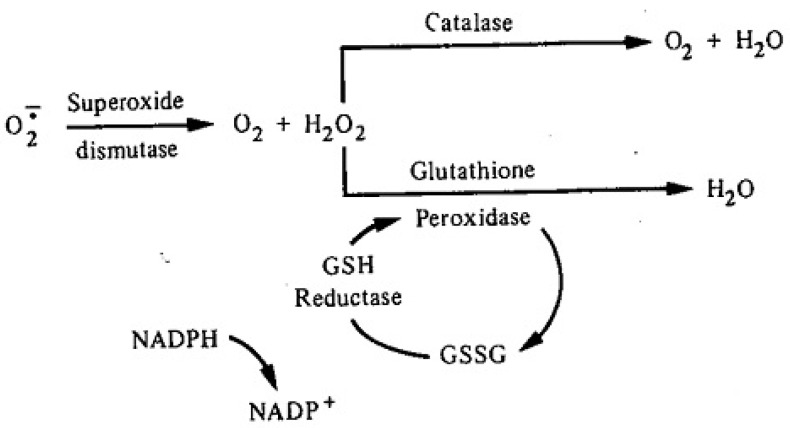
Schema Depicting Major Antioxidant Enzymes Involved in Maintaining Intracellular Redox Balance [22].

ROS are known to damage nucleic acids, proteins and lipids and are implicated in the pathophysiology of diabetes. Whereas ROS are generated in euglycemic, natural insulin-regulated glucose metabolism (Figure 2), levels are regulated by factors that regulate cellular respiration, e.g., the availability of NAD-linked substrates, succinate, and oxygen, and antioxidant enzymes that maintain the cellular redox balance. The latter include super oxide dismutase (SOD), which is a metalloprotein that dismutes superoxide anion to hydrogen peroxide. Other important enzymes are catalase and peroxidases. Catalase and glutathione (GSH) peroxidase reduce hydrogen peroxide to water. Oxidized glutathione (GSSG) is re-reduced to GSH by glutathione reductase in the presence of NADPH [21,22]. A simplified depiction of these reactions is shown in Figure 3. Under normal reduced conditions, only about 1–2% of the total oxygen consumption results in the formation of superoxide anion and hydrogen peroxide [19].

**Figure 4:**
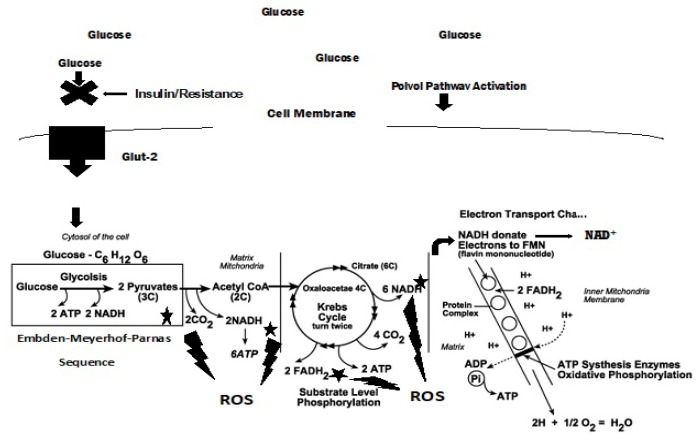
Hyperglycemic, insulin-resistant glucose metabolism. Although ROS are shown being formed at the starred sites of glucose metabolism, the actual formation of ROS occurs as electrons are being transferred from NADH and FADH_2_ down the ETC through complexes I, II, III and IV.

However, if the respiratory chain is highly reduced, as under hyperglycemic conditions, or if reduced cofactors accumulate, about 10% of the respiratory oxygen consumed may be lost as ROS [19]. This is depicted in Figure 4.

## 4. Electron Transport Chain, ROS Production, and Proton Pump Potential

Superoxide is released only into the matrix of the mitochondria when electrons are transferred through Complexes I and II of the ETC [24]. NADH formed in glycolysis and the Krebs cycle are oxidized in Complex I (**ubiquinone oxidoreductase**) that is composed of *NADH dehydrogenase*, FMN and iron–sulfur (Fe-S) clusters [25]. As electrons are transferred from Fe-S to coenzyme Q, four hydrogen ions pass from the mitochondrial matrix to the intermembrane space, thus increasing the proton motive force (Δ*p*) [26]. This occurs in a reasonably tightly coupled phosphorylated-oxidative (P/O) system. Interestingly, superoxide formation is strongly dependent on Δ*p,* and mitochondrial P/O uncoupling has been considered as a cytoprotective strategy in diabetes by reducing mitochondrial superoxide formation [26].

Complex II, **succinate dehydrogenase**, accepts electrons from succinate oxidation in the Krebs cycle and transfers two electrons to FAD. In turn, FADH_2_ transfers these electrons to Fe-S and then to coenzyme Q (ubiquinone). This process is similar to Complex I except that no protons are translocated into the intermembrane space.

Complex III, **cytochrome c reductase**, is composed of cytochrome b, Rieske proteins that contain two Fe-S clusters, and cytochrome c [27]. The latter can only accept a single electron at a time, and the process occurs in two steps; the Q cycle that produces superoxide has been comprehensively described [24]. The result is that Complex III releases four protons into the intermembrane space, thus contributing to Δ*p*, and it transfers the electrons, one at a time, to Complex IV [28].

Complex IV, **cytochrome c oxidase**, is the final electron carrier in aerobic cellular respiration and catalyzes the transfer of electrons to dioxygen to yield H_2_O [28].

## 5. Polyol Pathway

**Figure 5:**
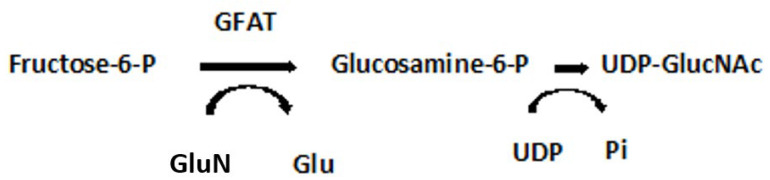
Hexosamine Pathway. GFAT, glutamine:fructose-6-phosphate-aminotransferase; UDP-GlucNAc, Uridine-5-diphosphate-N-acetylglucosamine c; GlutN, Glutamine; Glu, Glutamic acid; UDP, Uridine 5′-diphosphate; Pi, inorganic phosphate.

Persistent hyperglycemia leads to the activation of the Polyol pathway as depicted in Figure 4. It is estimated that around 30% of glucose in the body is metabolized through this pathway, leading to a NADH/NAD+ redox imbalance that contributes to oxidative stress in diabetes [29]. The first of the two-step reaction is glucose reduced to sorbitol that is then oxidized to fructose, depicted here:
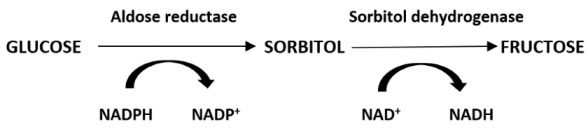


As aldose reductase reduces the high levels of intracellular glucose to sorbitol, the reaction consumes NADPH, which is a cofactor that is essential for regenerating the critical antioxidant, reduced glutathione (refer to Figure 3). Decreasing the level of reduced glutathione increases intracellular oxidative stress potential [29]. Increased ROS production from the polyol pathway contributes to diabetes complications [30]. The loss of glutathione found in the ocular lens under hyperglycemic conditions, the accumulation of sorbitol leading to the faster development of cataracts, and increased osmotic stress due to sorbitol accumulation demonstrate that the polyol pathway plays an important part in the pathophysiology contributing to diabetic complications [31,32]. Furthermore, also contributing to NADH/NAD^+^ redox imbalance resulting from the Polyol pathway are two NAD^+^ degradative pathways [33]. A mitochondrial family of signaling proteins (Sirtuins), histone deacetylase, and an ADP ribosyl transferase are NAD^+^ dependent and couple nutrient status, e.g., hyperglycemia, with stress responses—obviously impacting the redox balance as shown here [33,34]:
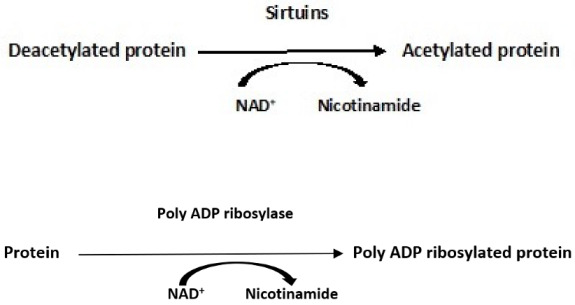


Indeed, it has been demonstrated in lymphatic muscle cells (LMC) that insulin resistance impairs glucose uptake, mitochondrial function, oxygen consumption rates, glycolysis, and ATP production [35]. As expected, acute insulin treatment activated insulin signaling, increased glucose uptake, and increased intracellular ATP in these cells. However, in insulin-resistant LMC, mitochondrial respiration, glycolysis, and ATP decreased. The decreased glucose uptake observed in insulin-resistant cells was, in part, attributable to changes in glucose transporter, **GLUT4**, translocation, the latter impaired by insulin signaling pathways [23]. Insulin-resistant LMC exhibited a significantly higher level of mitochondrial superoxide as the major source of ROS in these cells. As glucose uptake and glycolysis in these insulin-resistant cells was decreased, the elevated ROS levels suggest that activation of the polyol pathway is initiated as well as an increased flux through the **hexosamine pathway,** as depicted here (Figure 5) [30,36]:

UDP-GlucNAc is the precursor of all amino sugars required for the synthesis of proteoglycans, glycolipids and glycoproteins. GFAT regulates flux through the hexosamine pathway and is thought to be *causally* involved in diabetic nephropathy [36].

## 6. Protein Kinase C (PKC) Pathway and Cell Signaling

Intracellular ROS can be produced on the inner and outer sides of the mitochondrial inner membrane as electrons are being transferred down Complex III of the ETC—allowing them to act more easily as signaling molecules than those produced in the matrix [24]. Hyperglycemia causes repeated acute changes in cellular metabolism that ultimately result in diabetic tissue damage. As many of these tissues’ cells, e.g., ocular cells, are insulin independent, the response to hyperglycemia-induced signaling is complex and tissue specific. Thus, we limit our discussion here to cellular metabolism that is insulin dependent that occurs in mitochondrial respiration and its effect on ROS production.

**Figure 6:**
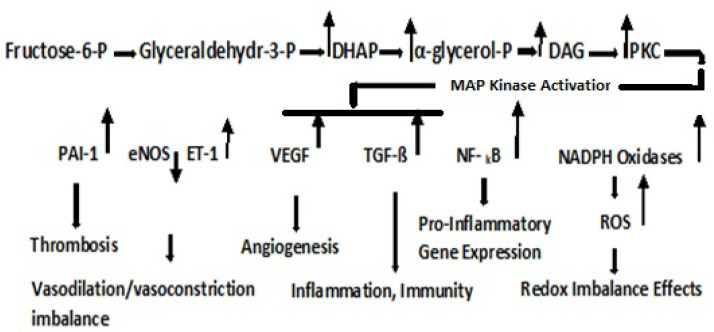
PKC Pathway and Pathogenic Effects. **DHAP**, dihydroxyacetone phosphate; **DAG**, diacylglycerol [41]; **PKC**, Protein Kinase C. Diagram depicts effects of isoforms ß and ð, which are both activated by DAG. The upregulation of **PAI-1**, Plasminogen activator inhibitor-1 (serpin E1), is a risk factor for thrombosis and atherosclerosis [40]; the downregulation of **eNOS**, Endothelial nitric oxide synthase, leads to vasodilation and upregulation of **ET-1**, Endothelin-1, a vasoconstrictor [42]; upregulation of **VEGF**, Vascular endothelial growth factor (a glycoprotein), mediating retinopathy and nephropathy [43]; **TGF-ß**, Transforming growth factor-beta (a multifunctional cytokine) that is pro-inflammatory and affects host immunity [44]; **NF-kß**, Nuclear factor-kappa B, a DNA binding protein factor required for the transcription of pro-inflammatory gene expression [45]; **NADPH Oxidase**, nicotinamide adenine dinucleotide phosphate oxidase (a flavocytochrome B heterodimer), a major source of ROS [46].

Glucose-6-P metabolism may be diverted to the pentose phosphate shunt to yield fructose-6-P when the rate of reoxidation of NADPH is limited. The metabolism of fructose-6-P, the starting point of the PKC pathway, is depicted in Figure 6. This results in the upregulation of dihydroxyacetone phosphate **(DHAP**, **α-glycerol-P**, and diacylglycerol, **DAG**). PKC comprises about 2% of the human kinome [37], and although its effects are cell type specific, it is generally activated by the formation of DAG [38]. The DAG precursor, α-glycerol phosphate, is the obligating acyl acceptor for lipogenesis in adipose tissue [39]. Long-term activation of the PKC pathway involves the DAG precursor, α-glycerol phosphate that is the obligating acyl acceptor for lipogenesis in adipose tissue [40]. Long-term activation of the PKC pathway involves translocation to the cell membrane. It plays an important role in the immune system through the activation of NF-kB, which is an important mediator of vascular permeability and is associated with hyperglycemia in diabetes. It also plays a pivotal role in cell regulation through the activation of a protein cascade, leading to the activation of mitogen-activated kinases (MAP Kinases). The PKC pathway with physiological consequences is depicted in Figure 6.

**Figure 7:**
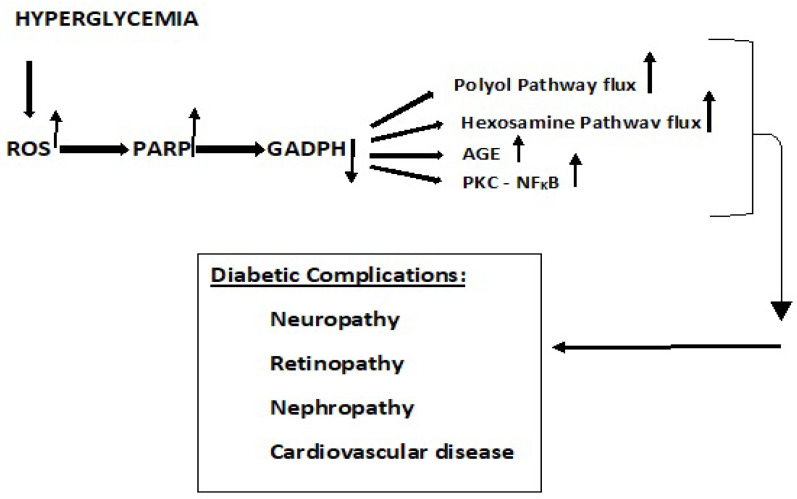
The influence of hyperglycemia on ROS level and activation of polyol pathway, hexosamine pathway, advanced glycation end products, and the PKC pathway, leading to tissue damage and diabetic complications [30]. Acute or chronic hyperglycemia upregulation of ROS production elevates PARP levels and downregulates GADPH levels. The latter activates and upregulates polyol and hexosamine pathways, accelerates the formation of AGE and activate PKC. These actions increase oxidative stress and ultimately impact the diabetic complications depicted.

Increased metabolic ROS activates poly (ADP-ribose) polymerase (**PARP**) that impacts the pathophysiology of diabetes [30,47]. PARP acts to decrease glyceraldehyde-3-phosphate dehydrogenase (**GADPH**) that results in an increase in methylglyoxal, a powerful glycating agent that is an important source of advanced glycation end-products (**AGE**) that contribute to diabetic complications [30,38]. ROS are generated when AGE are bound to receptor sites (**RAGE**). The overall role of these pathways and their resultant impact on diabetic complications are depicted in Figure 7 [30].

## 7. Lipid Oxidation, Peroxidation, Inflammation, and Immunity

**Figure 8:**
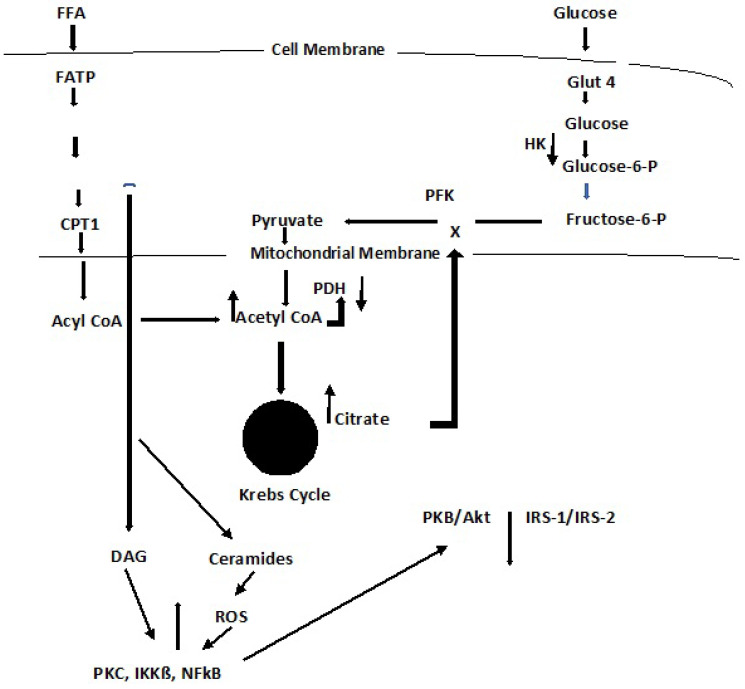
Lipid Oxidation and Cell Signaling [52]. FFA, free fatty acid; FATP, free fatty acid transport protein; FACS, free fatty acid acyl-CoA synthase; CPT1, carnitine palmitoyl transferase 1; DAG, diacylglycerol (refer to Figure 5); PDH, pyruvate dehydrogenase; PFK, phosphofructokinase; HK, hexokinase; GLUT 4, glucose transporter 4; PKC, protein kinase C; IKKß, inhibitory kß kinase ß; NFkß, nuclear factor kß; P13K, phosphoinositide 3-kinase; PkB/Akt, protein kinase B; IRS-1/IRS-2, Insulin receptor substrate 1 and 2.

Dyslipidemia is a hallmark of DM and a major risk factor for CVD [9,30,48]. The clinical manifestations of dyslipidemia include high plasma triglyceride levels, low HDL cholesterol, and increased plasma VLDL. The lipid changes associated with T2DM are thought to be a consequence of systemic free fatty acid (**FFA**) flux secondary to insulin resistance [48,49]. The relationship between FFA regulation and gluconeogenesis in diabetes has been elusive. It has been shown that at least 35% of gluconeogenesis in T2DM patients is FFA dependent [50]. An early effort to explain the competitive oxidation between glucose and fatty acids resulted in what became known as the glucose–fatty acid cycle, or Randle cycle [51]. This cycle was based on two lines of evidence. First, there is evidence for changes in the rate of release of FA under different experimental conditions, and second, there is evidence that FA induces changes in carbohydrate metabolism in normal animals similar to those seen in muscle tissue in DM. A schematic of the cycle is shown in Figure 8 [52]. FFA are transported across the cell membrane by fatty acid transport protein 1 (**FATP1**); a fatty acid acyl-CoA synthase (**FACS**) yields **Acyl-CoA**. These reactions occur in the cytosol of the cell. Acyl-CoA is transported across the mitochondrial membrane by carnitine palmitoyl transferase 1 (**CPT1**).

Acyl-CoA induces ß-oxidation in both the cytosol and the mitochondria. The former promotes gluconeogenesis. In the mitochondria, ß-oxidation results in an upregulation of acetyl-CoA that feeds into the Krebs cycle, increasing citrate that inhibits hexokinase and pyruvate dehydrogenase activities. The hexokinase inhibition results in the downregulation of glucogenesis. It also results in the formation of FADH_2_ and NADH that yields ROS as electrons are passed through the ETC.

As with other pathophysiological responses to insulin resistance and T2DM, dyslipidemia has been shown to be both complex and cell specific. Thus, several deviations from the glucose–fatty acid cycle have been reported, and the influence of cell signaling may hold the key to some of these varied responses [52]. As depicted in Figure 8, cellular Acyl-CoA leads to the metabolism of diacylglycerol (**DAG**) and ceramides. DAG activates PKC, IKKB, and NFkB that act to suppress PKB/Akt and IRS-1/IRS-2. IRS-1 inhibition decreases insulin-stimulated glucose transport, GLUT 4, and glucose uptake [52]. Ceramide increases free radical generation that, in turn, downregulates Akt, and GLUT 4 as well as causes insulin resistance [53].

Acyl CoA formation in the cytosol, or within the mitochondria, both may stimulate the ß-oxidation of fatty acids [52]. Beta-oxidation in the cytosol occurs in the peroxisome and upregulates the glyoxylate cycle through which glucose is synthesized. Beta-oxidation in the mitochondria results in 2-carbon acetyl-CoA that enters the Krebs cycle, producing ATP and NADH; the latter may increase ROS through the ETC [54]. A general depiction of these reactions is seen in Figure 8. A generalized summary reaction of ß-oxidations is shown here:

**R (16) CH_2_-COOH + acyl-CoA → 8 acetyl-CoA → 8 FADH_2_ + 8 NADH**

The equation depicts a 16-carbon saturated fatty acid undergoing complete ß-oxidation with two carbon fragments reacting with **acyl-CoA** to form eight acetyl-CoA molecules that enter the Krebs cycle. Complete oxidation yields eight **FADH_2_** and eight **NADH** that, under hyperglycemic conditions, produce ROS in the ETC.

It has been known that under hyperglycemic conditions, as in T2DM, elevation in circulating triglycerides and free fatty acids occurs that represent a serious dysfunction in lipid dynamics [55]. Thus, lipid oxidation plays a significant role in the development of DM.

Polyunsaturated fatty acids (PUFA) are particularly susceptible to free radical attack. The radical, e.g., a hydroxyl radical, attacks a PUFA to yield a PUFA radical (PUFA^•^) that, in turn, reacts with molecular oxygen to form a PUFA peroxy radical (PUFA-O-O^•^). The latter, by hydrogen abstraction from an adjacent PUFA, yields an unsaturated hydroperoxide (PUFA-O-OH). In addition, the reactions of lipid peroxidation show how the newly formed PUFA^•^ radical propagates a chain reaction that results in extensive disruption of the cell’s membranes. Persistent disorder and imbalance of free radical lipid oxidation products, as manifested by lipid hydroperoxides, leads to membrane dysfunction and disease development. It has been shown that diabetic patients with higher blood glucose level had an association with free radical-mediated lipid peroxidation [56]. There was also an imbalance in oxidant and antioxidant systems in T2DM. Such an imbalance between lipid hydroperoxide (LHP) level and decline in antioxidant activity (AOA), as reflected in the LHP/AOA ratio, has been shown to reflect the progress of disease [57]. Lipid peroxidation has been positively associated with complications occurring in T2DM and with systemic inflammation. Increased lipid peroxidation aggravates systemic inflammation [58].
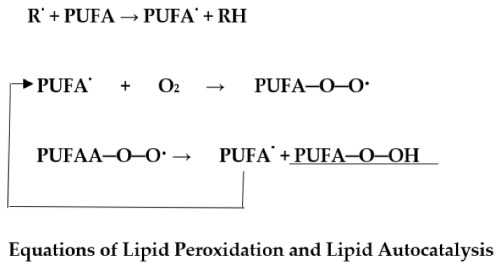


**Figure 9:**
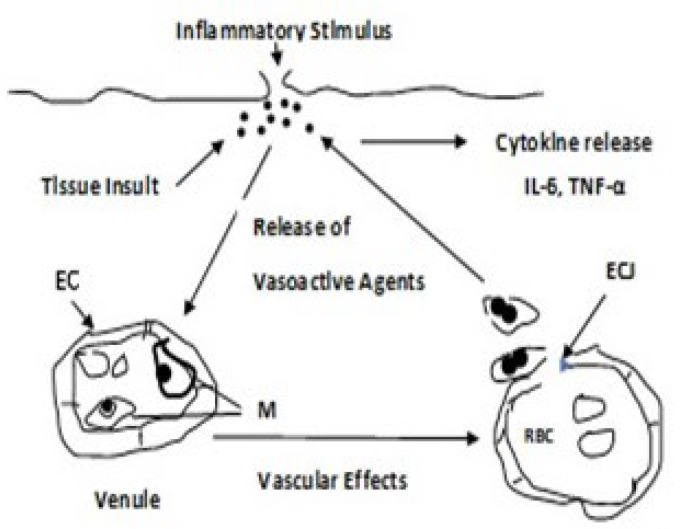
Acute Inflammatory Response. EC, endothelial cells; M, monocyte/macrophage; RBC, red blood cell; ECJ, endothelial cell junction. Upon tissue insult, several vasoactive and chemotactic substances are released at the injury site. The vasoactive substances act upon the endothelial cells of the venule, producing vascular effects. The activated macrophages escape through the endothelial cell junctions and are attracted to the inflammatory site where cytokines are released [60,61,62].

**Figure 10:**
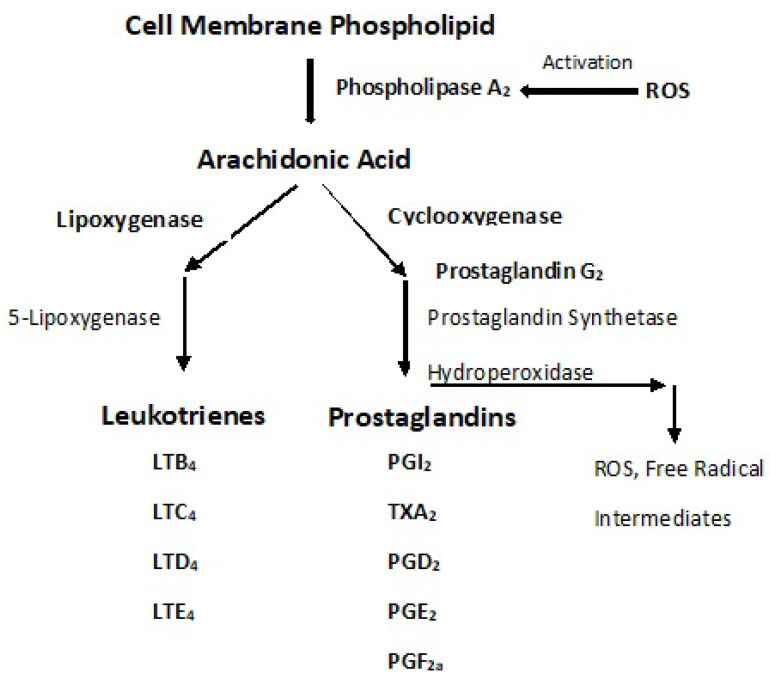
Pro-Inflammatory Leukotrienes (LT) and Prostaglandins (PG) Metabolized from Arachidonic Acid (AA). Isoforms of phospholipase A_2_ have been reported to be activated by ROS [63]. phospholipase A_2_ cleaves AA from cell membrane phospholipids. AA is oxidized through the lipoxygenase pathway to yield leukotrienes, eicosanoid inflammatory mediators, which are released by macrophages and are involved in the inflammatory reactions [64]. LTB_4_ is a potent chemotactic agent. The prostaglandins are derived from AA and metabolized through the cyclooxygenase pathway. They are involved in the inflammatory response as well as other pathogenic mechanisms [65]. PGE_2_ is a potent pro-inflammatory PG and is involved in all the signs of inflammation, although it also can exert anti-inflammatory effects during neuroinflammation.

Whereas the mechanism(s) by which chronic inflammation promotes the development of T2DM is unclear, adipose tissue synthesizes pro-inflammatory cytokines, e.g., interleukin (IL)-6, IL-1, and tumor necrosis factor TNF-α, indicating that inflammation and innate immunity are etiological factors in the pathogenesis of T2DM [59]. Furthermore, several vasoactive agents and immunopotentiators, important participants in the inflammatory process, are dependent on PUFA peroxidation (Figure 9 and Figure 10) [60].

As ROS is a mediator for the activation of pro-inflammatory signaling pathways; hyperglycemia-induced ROS production favors the induction of M1-like pro-inflammatory macrophages at onset and during the progression of DM [61].

It is evident that dyslipidemia is a major factor in the pathophysiology in T2DM and that the overproduction of ROS is directly, or indirectly, responsible for many of the subsequent clinical complications. Under hyperglycemic conditions, there occurs an increased flux of FFAs that are oxidized by the mitochondria. When acetyl CoA is derived from the ß-oxidation of FFA and oxidized in the Krebs cycle, it leads to NADH and FADH_2_ formation and the overproduction of ROS. Polyunsaturated lipids are particularly subject to attack by radicals, e.g., hydroxyl radical, leading to their peroxidation. Lipid peroxidation is associated with systemic inflammation. Activated macrophages are attracted to inflammatory sites where a respiratory burst occurs, and cytokines are released. As ROS is a mediator for activation of pro-inflammatory signaling pathways, hyperglycemia-induced ROS production favors the induction of pro-inflammatory macrophages at onset and during the expression of DM. ROS also activates some isoforms of phospholipase A_2_ that cleave arachidonic acid (AA) from the cell membrane phospholipid. AA is then oxidized through the lipoxygenase and cyclooxygenase pathways to form pro-inflammatory leukotrienes and prostaglandins as well as ROS and free radical intermediates.

## 8. Some Complex Considerations

Mitochondrial uncoupling proteins (UCP) catalyze a regulated proton leak across the inner mitochondrial membrane without the generation of ATP [66]. Five UCP have thus far been identified, each with specific functions [67]. Superoxide activates these mitochondrial uncoupling UCP [68]. UCP-1 can also be activated by long-chain FA and inhibited by purine nucleotides, e.g., ATP. UCP-2 and UCP-3 inhibit ROS production. Indeed, chronic hyperglycemia results in an upregulation of UCP-2 that reduces ROS production, resulting in reduced ATP production and insulin secretion that is consequently expressed as T2DM [67]. As chronic inflammation is associated with excessive ROS production in diabetic vasculopathy, UCP-2 may play a protective role by inhibiting excessive ROS formation. UCP-2 can also regulate FA and lipid metabolism related to obesity, which is an independent risk factor for DM. An interesting confluence of theories arise here. One pertains to chronic inflammation playing a crucial role in the development of obesity-related insulin resistance and T2DM [69]. The second involves the downregulation of ETC genes in visceral adipose tissue in T2DM that is independent of obesity [70]. Both evoke TNF-α. The first proposes that chronic inflammation is partially responsible for insulin resistance in T2DM. The second proposes that the downregulation of oxidative phosphorylation genes occurs, in part, by TNF-α regulation of ETC gene expression in visceral adipose tissue. The role of UCP-2 in affecting signaling pathways that lead to inflammation and vasoconstriction by reducing ROS may be a crucial factor not considered in these two theories. UCP must be considered in the complex milieu of metabolic reactions reflected in DM.

Insulin receptor (IR) is a membrane-bound glycoprotein found in all mammalian cells and functions in transmembrane insulin signaling. There are two known isoforms [71]. Although there is a single gene (INSB) expressing IR, alternative splicing produces the two isoforms. IR is primarily concerned with maintaining glucose homeostasis. When activated, IR induces glucose uptake. Insulin resistance represents a decrease in insulin signaling and results in hyperglycemia and T2DM (see Figure 7) [52]. Mutations in the IR gene or alterations in the abundance of the two isoforms would be expected to contribute to insulin resistance, glucose uptake, and, indirectly, influence FA uptake.

These factors must be interwoven into the complex milieu of metabolic and signaling responses represented in the pathophysiology of T2DM.

## 9. Antioxidants

The various pathways by which ROS are generated have been discussed, as has the overwhelming of the scavenging abilities of natural antioxidant defenses. An obvious approach to upgrade ROS defenses would be through supplementation with antioxidants. Among the antioxidants derived from the diet that have been considered for a therapeutic role are vitamins C, E, and carotenoids [72]. There have been mixed results with vitamin E showing no effect and vitamin C producing positive effects on diabetic complications. However, supplemental vitamin C has been associated with increased risk of CVD mortality in postmenopausal women with diabetes [73]. Vitamin C, like carotenoids, may, under certain conditions, act as a pro-oxidant [74].

Epidemiological studies have suggested that diets rich in polyphenols are associated with a significant reduction in risk for developing T2DM [75]. Aside from antioxidant activity, some polyphenols decrease glucose uptake and may attenuate postprandial hyperglycemia. Results from studies of specific polyphenols are still controversial.

Flavonoids are polyphenolic compounds, of at least 6000 phenolic compounds, found in fruits, vegetables, teas, cocoa and chocolate [76]. Flavonoids exert their anti-diabetic effects by targeting cellular signaling pathways in specific tissues, influencing ß-cell function, insulin sensitivity, glucose metabolism, and lipid profile [77].

*N*-Acetylcysteine (NAC) is a stable form of l-cysteine that is necessary to produce glutathione, which is a major antioxidant. NAC has been reported to be effective in reducing diabetic complications—presumably by affecting glucose homeostasis and reduction in ROS production [72]. However, in hyperglycemic T2DM patients receiving NAC supplementation, no benefit on markers of glucose metabolism, ß-cell response, or oxidative status was observed, and it was concluded that NAC supplementation was unlikely to be a valuable therapeutic approach [78]. It has been reported that when glycine was added to the NAC supplement (GlyNAC) and provided to T2DM patients, GlyNAC improved mitochondrial dysfunction, insulin resistance, improved glutathione synthesis, and lowered oxidative stress. [79].

Generally, antioxidant supplementation of T2DM patients have been less than encouraging. Brownlee [30] posits that to reduce direct oxidative damage, the amount of superoxide must be reduced and that conventional antioxidants are unlikely to do this effectively. Conventional antioxidants neutralize ROS on a one-for-one basis, whereas hyperglycemia-induced ROS is a continuous process. He suggests a new type of antioxidant, a catalytic antioxidant such as an SOD/catalase mimetic that works continuously. While not acting to reduce superoxide directly, GlyNAC does work continuously to regenerate glutathione to help restore the redox balance.

## 10. Discussion and Conclusions

An online search of published papers on diabetes lists slightly over 49 million in the past 30 years with over two million being research papers. The current synopsis is obviously not a comprehensive review but aspires to serve as a primer of how and where ROS are formed in the cell and their role in the etiology and pathophysiology of DM. Most remarks refer to T2DM as it comprises about 95% of all DM and is the fastest developing disease globally. This effort draws heavily upon the herculean undertaking of Michael Brownlee in presenting an “unifying mechanism” for the pathobiology of diabetic complications in the Banting Lecture of 2004. Whereas the cited literature in this synopsis generally supports the underlying basis of a unified approach to our understanding of the pathogenesis of DM, it must be emphasized that exceptions to this generalized approach have been reported and that diabetic pathophysiological responses are cell and tissue specific.

Oxidative stress, via the production of ROS, is characterized by an imbalance between the generation and removal of these radicals. However, under hyperglycemic conditions, or if reduced respiratory cofactors accumulate, it is estimated that around 10% of the respiratory oxygen consumed may be lost as ROS. Superoxide anion is the major radical formed and occurs when electrons are transferred from reduced cofactors NADH and FADH_2_ through Complexes I and II (and the Q cycle of Complex III) of the ETC. Persistent hyperglycemia leads to the activation of four of the pieces of Brownlee’s puzzle, i.e., the polyol pathway, the hexosamine pathway, the protein kinase C pathway and to the formation of advanced glycation end products. The upregulation of hyperglycemic-induced ROS production downregulates glyceraldehyde-3-phosphate dehydrogenase that activates the polyol, hexosamine, and PKC pathways and accelerates the formation of advanced glycation end products, all of which contribute to diabetic complications and impact the redox balance.

Dyslipidemia is a hallmark of DM and a major risk factor for CVD. The lipid changes associated with T2DM result as a consequence of systemic FFA flux that results subsequently in insulin resistance. An FA acyl-CoA synthase yields acyl-CoA that induces ß-oxidation that, in turn, yields higher levels of Acetyl-CoA. The latter feeds into the Krebs cycle, increasing citrate that inhibits hexokinase and pyruvate dehydrogenase activities. Hexokinase inhibition downregulates glucogenesis. ß-oxidation in the mitochondria results in the formation of FADH_2_ and NADH that yields ROS as electrons are passed through the ETC. The ROS attack of PUFA leads to lipid peroxidation and autocatalysis. Increased lipid peroxidation aggravates systemic inflammation. Adipose tissues synthesize pro-inflammatory cytokines, particularly IL-6, Il-1, and TNF-α, indicating that inflammation and innate immunity are etiological factors in the pathogenesis of T2DM. Several vasoactive agents and immunopotentiators involved in the inflammatory process are dependent on PUFA peroxidation. ROS also activates phospholipase A_2_ that cleaves AA, which is then oxidized through the lipoxygenase and cyclooxygenase pathways, producing pro-inflammatory leukotrienes and prostaglandins, as well as ROS and other radical intermediates. It is evident that the lipid oxidation of FFA and lipid peroxidation are major factors in the pathophysiology of T2DM and that the overproduction of ROS is directly, and/or indirectly, responsible for many of the subsequent clinical complications.

Whereas antioxidant therapy remains as a potential therapy, or co-therapy with DM drugs, the root of the problem lies with obesity and sedentary lifestyle. The more obese and sedentary an individual, the greater the degree of insulin resistance and dyslipidemia—hallmarks of T2DM and MetS [80]. Introducing changes in diet and lifestyle is a tough nut to crack!
